# Edvard Munch (1863-1944). Self-Portrait After the Spanish Flu (1919-20)

**DOI:** 10.3201/eid0903.AC0903

**Published:** 2003-03

**Authors:** Polyxeni Potter

**Affiliations:** * Centers for Disease Control and Prevention, Atlanta, Georgia, USA

**Figure:**
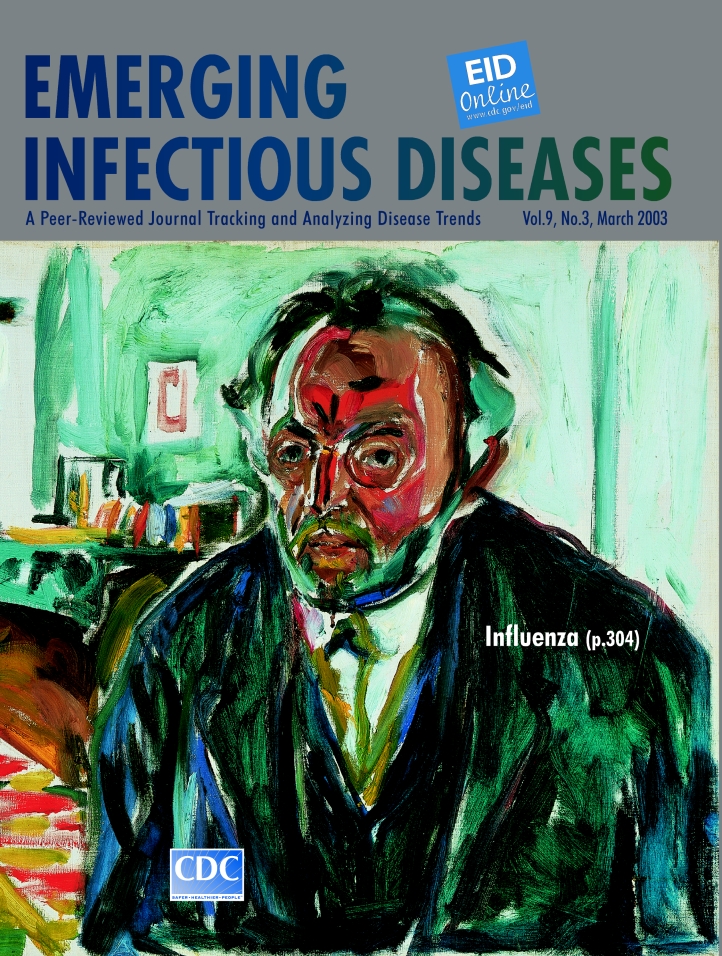
Edvard Munch (1863–1944) Self-Portrait after the Spanish Flu (1919) Oil on canvas 150.5 cm x 131 cm The Munch Museum/the Munch-Ellingsen Group/Artists Rights Society, New York

“Illness, insanity, and death…kept watch over my cradle and accompanied me all my life,” noted innovative Norwegian artist Edvard Munch. Deeply affected by the untimely death of his mother (when he was 5) and his 15-year-old sister (when he was 14), he devoted his early artistic efforts to painting their predicament and the ravages of tuberculosis, “…the wan face in profile against the pillow, the despairing mother at the bedside, the muted light, the tousled hair, the useless glass of water” (*1*). His own fragile physical and emotional state dominated the way he viewed and executed his art. In his middle years, incapacitated by depression, he spent time in a sanatorium in Denmark, and even though he recovered, his work never regained its initial expressiveness (*2*).

Munch studied in Oslo and traveled extensively to Italy, Germany, and France, where he took in the influences of his contemporaries (particularly Toulouse-Lautrec, van Gogh, and Gauguin) who were turning the angst of modern civilization into symbolism and stark expressionism (*3*). Preoccupation with decadence and evil pervaded the artistic and literary climate of the day. Darkness and horror inspired deeply personal, highly expressive art in a variety of styles, all of which fit under the umbrella of symbolism, as long as they embodied its peculiarly gloomy state of mind (*2*). The movement’s emphasis on inner vision rather than observation of nature captured Munch’s haunted imagination and engaged his moody genius.

Inspired by the work of Henrik Ibsen, Munch studied psychoanalysis and created art that unraveled the mysteries of the psyche. His canvases are filled with agonizing uncertainty and excruciating loneliness, anticipating Ingmar Bergman’s theater and cinematic work (*3*). His personal neuroses and physical ailments permeate the cultural anxiety expressed in his work. Even as he painted the existential drama of his own life, Munch did so without graphic depictions of monsters or apparitions. Rather, he provoked emotional response through unnatural color, internal rhythm, and undulating lines, as in The Scream, one of the most reproduced and universally acclaimed paintings in the history of art. Munch’s most ambitious (unfinished) work, The Frieze of Life, comprised a sequence of connected panels intended to expose the illusory nature of optimism and bring to public view the painter’s innermost feelings about life—from birth to death (*3*).

Pestilence, which traumatized Munch’s early years in the form of tuberculosis, continued to rule his life and fuel his journey through the vagaries of the human condition. In Self-Portrait after the Spanish Flu on this month’s cover of Emerging Infectious Diseases, the tormented painter appears judge and victim of this pandemic killer. The terse yet unsteady demeanor, the puffy discolored glare, the quivering lines of fever and chills, only highlight the despair and isolation of the “grippe” patient, the oppression, the weakness, the malaise, the lack of air, the stupor, the hopelessness.

Munch’s preoccupation with suffering in this self-portrait is fully understood by those who study the Spanish flu pandemic. Erupting during the final stages of World War I, this global disaster reinforced the era’s nihilism and apocalyptic visions of despair. “I had a little bird/Its name was Enza/I opened the window/And in-flew-enza,” morbidly sang the children as they skipped rope (*4*). Specimens from the remains of flu victims buried in permafrost provide some clues about the 1918–1919 strain. Highly contagious and unusually virulent, the deadly flu, circled the globe, taking its toll among the youngest and healthiest. Medicine was then only beginning to understand infectious diseases and to take modest steps towards diagnostics and therapy.

Infectious disease medicine has come a long way, yet Munch’s specter of the flu is alarmingly current. Surveillance of circulating viruses is increasing and flu vaccination has entered the mainstream, but epidemics are still frequent and strains arising from antigenic shift keep the next flu pandemic just around the corner.
